# Examining the effects of stroke on students’ L2-grit levels in an EFL context: A case of Northern Iraq

**DOI:** 10.3389/fpsyg.2022.1067901

**Published:** 2022-11-11

**Authors:** Diyar J. M. Mohammed, Behbood Mohammadzadeh, Yalın Kılıç

**Affiliations:** ^1^ELT Department, Faculty of Education, Cyprus International University, Nicosia, Turkey; ^2^Department of General Education, Cihan University Sulaimaniya, Sulaymaniyah, Iraq

**Keywords:** L2-grit level, stroke level, EFL context, university students, Northern Iraq

## Abstract

This article investigates the effects of stroke on students’ L2-grit levels in an EFL context in Northern Iraq. A model was created to find how various components such as verbal, non-verbal, valuing, and activities that determine stroke levels affect students’ grit. This study adopted the L2- Grit scale and a language domain-specific grit scale to measure the learners’ L2-grit levels. Also, the Students’ Stroke Scale (SSS) was used to measure the participants’ stroke levels. The study’s sample consisted of 199 participants from various academic levels, undergraduate, graduate, and postgraduate at several Northern Iraq universities. The results reveal a positive and statistically significant association between the score on the L2-Grit scale and the score on the Stroke scale; more specifically, as the scores on the L2-grit scale rise, so do the scores on the Stroke scale. The mean of low stroke is lower than the means of medium stroke and high stroke indicating that as people’s stroke levels grow, so does their L2-grit status. The regression coefficients estimated within the framework of the regression model structured with the logit, the link function, are the same in each category of the dependent variable, satisfying the parallel curves assumption. The overall results show that positive stroke helps learners’ L2-grit levels to arise and lead to a better learning process.

## Introduction

EFL and second language acquisition (SLA) receive a great deal of attention from language, and education, because of their crucial roles in furthering the learning and teaching agendas of many countries and institutions and psychology academics. In the north of Iraq, which has its own Kurdish Regional Government (KRG), students must take EFL courses from elementary school through university (Ismael and Mohammadzadeh, 2022). Consequently, it is essential to investigate the various factors contributing to improving English language learning and teaching.

Willingness to communicate (WTC) is the goal students of English as a foreign language work toward achieving. WTC in a second language has been the subject of extensive research, and the term “readiness to enter into discourse at a particular time with a specific person or persons, using a L2” has been developed to describe this ability (as cited in Lee, 2020, p. 1). On the other hand, one must not limit the concept of communication to merely verbal exchanges. Another vital talent that has garnered emphasis in recent years is the ability to communicate in writing. This is because written communication is used extensively in print and online and because teachers use it to evaluate their students’ progress (Yancey, 2009; Sedita, 2013).

Most of the time, a person’s talent and grades are the only elements considered when predicting that person’s future success, and many or all other individual factors are neglected. One cannot argue that skill is not vital in forecasting one’s success; yet, it should not be considered the only determinant of success, as many people with great talent fail to accomplish anything of worth in their lives. Their cognitive and non-cognitive skills substantially influence an individual’s degree of success or failure. Cognitive skills are referred to as the capacity for thinking, remembering, and concentrating. On the other hand, non-cognitive talents include motivation, the ability to self- regulate and believe in one’s capabilities, coping mechanisms, the ability to bounce back from setbacks, and personality.

Positive Psychology (PP) has become an essential subject in EFL and SLA due to its dedication and concern for the comfort and pleasure of the learners during the learning process (Gabryś-Barker et al., 2016; Oxford, 2016; Strzałka, 2016; Li, 2020). Positive psychology is categorised by Seligman and Csikszentmihalyi (2014) according to subjective, individual, and group levels. For the subjective level, Seligman and Csikszentmihalyi assert that it is about “valued subjective experiences: wellbeing, contentment, and satisfaction (in the past); hope and optimism (for the future); and flow and happiness (in the present)”; the individual level is about traits such as “the capacity for love and vocation, courage, interpersonal skill, aesthetic sensibility, perseverance, forgiveness, originality, future mindedness, spirituality, high talent, and wisdom”; the group level, “is about the civic virtues and the institutions that move individuals toward better citizenship: responsibility, nurturance, altruism, civility, moderation, tolerance, and work ethic” (p. 5). On the basis of the aforementioned levels, Gabryś-Barker et al. (2016) assert that subjective experiences, individual traits, and the learning environment are crucial factors in influencing the performance of EFL learners.

Grit is one of the positive non-cognitive attributes. The concept of “grit” as a distinguishing characteristic of one’s personality gained attraction in the field of psychology in 2007, when it was first presented by Duckworth et al. (2007). It is thought to be the only non- cognitive characteristic that all successful people have in common with one another. Duckworth et al. (2007) say that grit is perseverance and passion for long-term goals and that grit “entails working strenuously toward challenges, maintaining effort and interest over the years despite failure, adversity, and plateaus in progress” (p. 1087). The Department of Education of the United States of America has designated grit, along with tenacity and perseverance, to be one of the most critical predictors of success. This declaration is an essential acknowledgement of grit’s essential role (Shechtman et al., 2013). In spite of all the attention that grit has garnered over the course of the last decade, the EFL has yet to fully investigate it. According to MacIntyre et al.’s (2019) research, grit plays an important part in the process of language acquisition, and this aspect of the phenomenon needs to be investigated.

Researchers have only just begun to acknowledge the significance that grit and positive psychology have in enhancing a learner’s ability to pick up a new language. Consideration of these factors is a recent development. However, there are still some gaps in the existing research examining how the interaction between students and their professors is related to the students’ grit levels in an English as a Foreign Language setting. The current study investigates whether or not there is a significantly distinct relationship between the recognition that students in Northern Iraq receive inside and outside of the classroom and their levels of grit. Specifically, this paper is an attempt to answer the following research questions (RQ):

- RQ1: Is there a significant relationship between the Grit scale score and the Stroke scale score?
- RQ2: Do the Stroke scale scores of the individuals show a significant difference according to the Grouped Grit Status (Low, Medium, and High)?
- RQ3: Are there any effects of stroke components (verbal, non-verbal, valuing, and activities) on grit conditions?

## Literature review

### Grit

Research on grit, which is still in its infancy, has attracted a significant amount of focus in recent years. Duckworth et al. (2007), who defined grit as “passion and perseverance for especially long-term goals,” is one of the most prominent and well-known researchers on grit (p. 1087). According to Duckworth, the initial spark that got her interested in studying grit was when she questioned what elements, other than opportunity and talent, determine one person to be more successful than another person. “Grit does not just have resilience in the face of failure,” says Perkins-Gough, “but also having deep commitments that you remain loyal to over many years” (2013, p. 16).

Grit is an abbreviation that stands for the following four words: guts, resilience, initiative, and tenacity (Thaler and Koval, 2015). In order to be considered a person with grit, one must exhibit all four of these attributes. It was discovered in a study conducted by the United States Department of Education in 2013 that grit is equally as significant as intelligence in determining one’s level of achievement. Duckworth has developed a self-report grit scale that may be used to assess the grit levels of an individual. This will allow grit to be measured. In the Western context, it was discovered that grit had a positive correlation with learners’ achievement and retainment (Schmidt et al., 2017; Park et al., 2018; Clark and Malecki, 2019).

Later, Teimouri et al. (2020) developed a new grit scale based on Duckworth’s, which is utilised in this article. The grit scale proposed by Teimouri et al. (2020) is a domain-specific grit scale that measures the grit levels of SFL and EFL learners. According to Sudina and Plonsky (2021) perseverance of effort which is a “subscale of grit was a stronger predictor of students’ grades and self-reported proficiency” (p. 831). This demonstrates the significance of studies on grit and the development of its application in language acquisition, as students frequently quit when confronted with challenges.

### Stroke

The dynamic relationship that develops between a teacher and the students under their tutelage is of critical significance in teaching and learning environments. Students benefit from having positive relationships with their professors because it helps them learn, gives them opportunities to develop the required interpersonal skills, lowers their anxiety, and boosts their drive. Eric Berne developed a method called transactional analysis (TA) that is now considered to be one of the primary methods for analysing interpersonal relationships, “TA is a theory of personality and systematic psychotherapy for personal growth and personal change” (Stewart and Joines, 1987, p. 3). This approach has seen a great deal of application in the fields of counselling, education, communication, and psychology (Solomon, 2003; Barrow, 2007). In educational contexts, TA is utilised to aid in the establishment of clear communication between instructors and students and to prevent the initiation of confrontations that are counterproductive (Stewart and Joines, 1987). Ego states, transactions, life scenarios, life positions, temporal structures, and strokes are the six components that make up the TA method (Shirai, 2006).

The term stroke refers to any action taken to recognise the presence of another person or their ideals (Shirai, 2006). It is a measure of the human capacity for recognition. Interpersonal connections that can be seen as satisfying an emotional need are called strokes. There are many distinct varieties of strokes, including those that are verbal or non-verbal, positive or negative, conditional or unconditional. A simple greeting all the way up to an in-depth discussion might be considered verbal strokes. Non-verbal strokes include things like smiling, nodding your head, shaking hands, and other such actions. Positive strokes are experiences that the recipient views as being pleasant and satisfying, whereas negative strokes are experiences that the receiver views as being unpleasant and unsatisfactory. The actions that people engage in are referred to as conditional strokes, whereas the qualities that people possess are referred to as unconditional strokes (Stewart and Joines, 1987).

### Research on grit

The Department of Education of the United States of America stated in 2013 that grit is among the most crucial qualities in deciding one’s success in this 21st century. As a result, ever since the concept of grit was first brought up in the academic world, a wide variety of studies on the topic have been carried out. Chang (2014) conducted research at a private college in the Southern United States to investigate whether or not differences in grit levels are associated with differences in academic performance based on race and gender. The research was carried out on first-year students, 51% of whom were female and 49% were male, 55% of the students were white, 18% were Hispanic, 14% were Asian or Pacific Islander, and 9% were black. According to the findings of the study, grit does not have any bearing on the grades received by first-year students. It also demonstrated that differences in race or gender do not have an effect on one’s level of grit. Credé et al. (2017) concur with Chang’s findings that gender and race are non-factors in determining whether an individual is gritty.

In more recent studies (Ma et al., 2020; Whipple and Dimitrova-Grajzl, 2021) researchers looked at the effects of grit on male and female students and found that there were significant gender differences in the effects of grit. According to the findings of Ma, Ma, and Lan, higher levels of grit have a beneficial effect on both males and females with regard to the association between teacher autonomy support and social competence. On the other hand, a lower level of grit was found to be significantly associated with the male participants’ association but not with the females. The participants in this study consisted of 1,009 undergraduates from China. On the other hand, Whipple and Dimitrova-Grajzl found that grit has a positive effect on the college point averages of the male participants, but that it has no significant effect on the college point averages of the female participants.

According to the findings of some studies, the correlation between age and grit varies depending on the setting. A number of studies (Duckworth et al., 2007; Peña and Duckworth, 2018; Bliss and Jacobson, 2020) demonstrate that there is a significant relationship between age and grit. Other studies do not find a correlation between the two (Rhodes and Giovannetti, 2021). According to Duckworth and her colleagues, grit is not a fixed trait; rather, it shifts and develops over the course of one’s life, and it may become more robust as a person ages due to the fact that, in general, older people value their goals and the world around them more than younger people do. Peña and Duckworth demonstrated that there is a significant correlation between age and grit. Additionally, Bliss and Jacobson found that age and grit have a strong correlation with one another, and that the two of them can successfully predict one’s academic success. In spite of this, Rhodes and Giovannetti (2021) conducted a study on getting older and discovered that there is no significant correlation between grit and age. They claim that one’s level of grit is unaffected by the passage of time, from young adulthood to old age.

Credé et al. (2017) carried out a study on the previously published research on grit, and as part of that process, they examined 88 samples. They came to the conclusion that grit has a moderate relationship with performance and retention across a variety of academic disciplines. In addition to this, they discovered that grit could predict performance, albeit to a limited extent. According to the findings of Credé et al. (2017) grit is a factor that predicts retention in a manner that is comparable to that of the traditional factors that have been the focus in recent years, such as high marks and cognitive abilities.

### Research on stroke and grit

Academic research has not yet conducted an exhaustive investigation into how stroke affects grit levels. The current study was made possible by the publication of two significant studies in 2022. These papers prepared the ground for the current paper to be produced. To begin, Yuan (2022) investigated the ways in which increasing students’ exposure to stroke in the classroom could boost their levels of grit. China served as the location for this study’s setting. In order for Yuan to achieve his objective, he gathered information from a sample of 316 Chinese university students who were studying English as a foreign language. These students came from more than 30 cities across China. The students were given three different questionnaires to fill out: one about the teacher’s stroke, one about student rapport, and one about the students’ grit. The most important takeaways from the study, which were obtained through running regression, are that there are positive associations between stroke and its effects on learners’ grit, and that these associations are correlated positively with one another.

Second, Shen and Guo (2022) carried out a study in which they investigated the connection between the amount of respect and support that students receive from their teachers and the amount of grit that the students exhibit in their academic pursuits. Respect and support are two essential aspects of stroke, despite the fact that the topic of stroke was not directly addressed in this passage. This research was carried out in China, and the sample size consisted of 613 individuals who were studying English as a second language. Initially, the Spearman correlation was utilised, and it was found that teacher respect, teacher support, and grit were strongly correlated with one another. Grit was also found to be strongly correlated with respect for teachers. After that, multiple regression analysis was performed, and the results showed that the respect and support of teachers had a significant impact on the higher levels of grit displayed by the learners. This was determined based on the findings of the study.

## Model of the research

**Figure 1:**
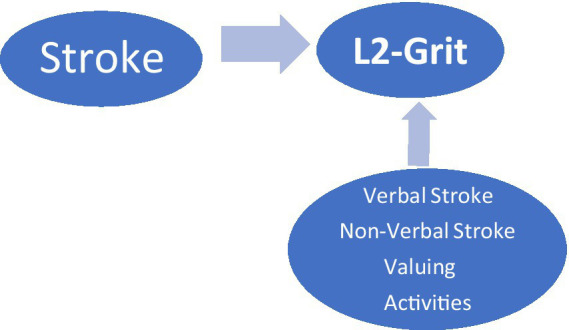
Model of the research.

Various studies show that stroke and grit levels are important in learning English. Examining the effect of stroke level on L2-grit status in the research model is the centre of the research. In this context, our study was designed within the scope of the research model in Figure 1. We conducted the study based on the components such as verbal, non-verbal, valuing, and activities that determine stroke levels included in the model.

## Theoretical framework

### Design

In this particular study, we carried out research in the form of a survey. We began by asking the participants some personal questions about their age, gender, city, university, and topic of study. These questions were presented in the form of a survey. Then, we utilised two scales to measure the L2-grit levels of the participants, derived from Teimouri et al. (2020), and the stroke status of the students using the SSS created by Pishghadam and Khajavy (2014). Survey research was used to “provide evidence on practise, attitudes, and knowledge” (Story and Tait, 2019).

### Participants and setting

Because there is a dearth of study conducted in this part of the world, we have decided to conduct our investigation in the northern region of Iraq. The concept of grit has been investigated in a variety of settings, including Iran, China, Japan, and Thailand (Yamashita, 2018; Gyamfi and Lai, 2020; Wei et al., 2020; Khajavy et al., 2021), and the purpose of our research was to make a contribution to the existing body of work on the topic. After obtaining the necessary clearance from the relevant authorities, the questionnaire was distributed to a number of educational institutions. Both instructors and students were requested to reply voluntarily to the survey. There was a total of 199 participants who responded to the survey, 108 of them were male and 91 of whom were female. There was a total of 146 participants who fell into the age range of 16–26, and there were 53 participants who fell into the age range of 26 and above. The age ranges of the participants were divided into two categories, 16–26 years old and 26 years old and above. The number of participants from private universities came in at 113, while the number of participants from public universities came in at 86.

## Instruments

The students’ cumulative grade point averages in their senior year of high school are taken into consideration when admittance decisions are made at governmental colleges and institutes in the Northern region of Iraq. The students will subsequently fill out a form titled “Zankoline” to indicate their desired departments. However, students must be careful when filling out this form; if they make a mistake, they may end up in a department they never selected. Traditional measurements such as this one is utilised in various nations, and they have proven to be fairly useful in determining students’ achievements (Kuncel et al., 2001). Recent research has demonstrated, however, that traditional measurements alone are insufficient; combining traditional and non-cognitive measures has proven to be far more accurate (Sternberg et al., 2012).

For students and learners of a language, frustration is a common experience. During the SLA process, one encounters a great number of challenges and roadblocks. When they cannot envision a successful future in their field, a lot of people give up, particularly students at SLA universities. Because of these challenges, the students and learners may experience feelings of depression and anxiety. Therefore, it will be necessary for them to have strong non-cognitive skills. Krashen (1992) presents the Affective Filter Hypothesis, which is a theory on non-cognitive skills (AFH). According to Krashen’s theory, the process of learning a language is directly influenced by the mental states of the learners, such as their levels of anxiety, motivation, and self-confidence. He continues by explaining that students who have a low level of fear, high levels of motivation, and high levels of self-confidence will be more successful in learning a new language.

### L2-grit scale

Numerous studies have been undertaken by researchers in order to identify the degree to which grit is related to other non-cognitive talents. Researchers Reraki et al. (2015) investigated the connection between motivation and grit in college students and came to the conclusion that there is a positive connection between the two concepts as well as a close connection between the two concepts. Changlek and Palanukulwong (2015) carried out an additional study on English language learners in Thailand, and similar to the previous research, they discovered a favourable link between motivation and grit.

According to Duckworth et al. (2007), there is a correlation between grit and the Big Five personality system. Neuroticism, extraversion, openness, agreeableness, and conscientiousness are the five characteristics that make up the Big Five, which Costa and McCrae (1992) define as the most dependable framework for describing personality. Mayer and Skimmyhorn (2017) conducted a study on two military cadets in order to establish whether or not grit corresponds with the Big Five. The participants in the study were required to complete challenging academic tasks. They came to the conclusion that grit has a very strong positive correlation with conscientiousness. According to the findings of the study, grit also had a favourable correlation with the other four Big Five factors.

Based on the understanding of grit and how effective it might be, the L2-Grit scale was used to measure the participants’ grit levels in this paper. In 2017, Teimouri, Plonsky, and Tabandeh worked together to develop a domain-specific grit scale for the language domain. Their work was inspired by the original grit scale developed by Duckworth et al. (2007), which was a domain- general grit scale. Teimouri et al. (2020) measured the motivational variables of intended effort, Second Language Willingness to Communicate, and attention. According to Lake (2013), concerning measuring intended effort related to real effort, the researchers have employed a six-point Likert-type scale consisting of three items. To measure the students’ willingness to communicate in L2 (L2 WTC), the researchers have used a six-point Likert-type four items scale adopted by Yashima (2002). L2 WTC refers to the students’ intention to use L2 when no one pushes them (Khajavy et al., 2018). Finally, to measure the students’ attention levels, the researchers have employed a six-point Likert-type three items scale.

Mindsets is another aspect that Teimouri et al. (2020) have explored. According to the Mindsets theory (Dweck and Yeager, 2019), there are two groups of people; those who possess fixed mindsets believe that their cognitive abilities are unchangeable, and those who possess growth mindsets believe that their cognitive abilities are flexible. In order to measure whether the students possess fixed or growth mindsets, the researchers have used a seven-point Likert-type four items. Teimouri et al. (2020) have used L2 anxiety and L2 joy for emotional measures. To measure the sample students’ L2 anxiety, they used a six-point Likert-type four items scale. Regarding measuring the L2 joy of the students, the researchers have employed a six-point Likert-type four items scale adopted from Teimouri (2017).

The final aspect of the sample students that Teimouri et al. (2020) have measured was language achievement. In order to measure the students’ language achievement, the researchers analysed three things; students’ grades in three English courses, their GPA (grade point average), and English language proficiency.

In the first question, Teimouri et al. (2020) wanted to learn how their newly-created L2-Grit scale is reliable and valid. The scale was valid and reliable as it was correlated to the domain-general Grit scale. Also, to further show its validity, the students’ self-report grit scale correlated with the teachers’ report on the students’ grit levels. Furthermore, the L2-Grit scale positively correlated to the students’ language achievement measures. The second question Teimouri et al. (2020) addressed in their research was the relationship between students’ motivation and emotions with their L2 grit. They have found out that motivation and emotions positively correlate to grit; gritty students were more inclined to learn, focus and engage in L2 classes. It is also shown that grit is negatively correlated to anxiety, similar results as (Changlek and Palanukulwong, 2015), but positively correlated to joy in L2 learning, a similar finding to (Credé et al., 2017). The findings also showed that the newly-created L2-Grit scale is more potent than the domain-general scale in predicting the relationship between the students’ motivation and emotions to grit.

Concerning the relationship between the students’ intelligence and grit, the findings of Teimouri et al. (2020) have shown that gritty students view a growth mindset to be the preferable type of intelligence. It has also shown a positive correlation between grit and a growth mindset and a negative correlation to a fixed mindset. This finding reiterates the idea put forth by Perkins-Gough (2013) that since grit contains perseverance of effort, then it is natural for gritty students to view a growth mindset of intelligence positively. Finally, regarding the third question of Teimouri et al. (2020), the research showed that grit correlates positively with language achievement. The research has also shown that the L2-Grit scale, a language domain-specific grit scale, is more accurate in measuring the relationship between grit and language achievement than the general- domain grit scale.

### Students’ stroke scale

Pishghadam and Khajavy (2014) created and validated a measure of student stroke in order to investigate the relationship between student stroke and motivation, which led to the discovery of a positive connection between them. Irajzad et al. (2017) were the ones in charge of another study that looked at the strokes that were derived from Iranian high school students who were enrolled in language classes. The findings revealed that teachers employ a wide variety of pedagogical approaches when working with students on the development of their language skills. They mostly attributed their success to the kinds of frameworks that they put in place during their professional lives.

## Findings

*Research Question 1:* Is there a significant relationship between the Grit scale score and the Stroke scale score?

Table 1 shows a moderate, positive and significant relationship between the Grit scale score and the Stroke scale score (*r* = 0.35, *p* < 0.01). This finding suggests a strong correlation between the Grit scale scores and the Stroke scale scores. Considering the coefficient of determination (*r^2^* = 0.12), it can be said that 12% of the total variance (variability) in the Grit scale score is due to the Stroke scale score (Table 2).

**Table 1:** Correlation between grit scale score and stroke scale score.

		Grit	Stroke
GRIT	Pearson correlation	1	0.350
	Sig. (2-tailed)		0.000
	*N*	199	199
STROKE	Pearson correlation	0.350	1
	Sig. (2-tailed)	0.000	
	*N*	199	199

Correlation is significant at the 0.01 level (2-tailed).

**Table 2:** ANOVA results of individuals’ stroke scale scores according to grouped grit status (low, medium, high).

Source of variance	Sum of squares	Df	Mean square	*F*	*P*	Significant difference
Between groups	10.05	2	5.02	12.94	0.000	Low-Medium
Within groups	76.07	196	0.39			Low-High
Total	86.12	198				

*Research Question 2:* Do the Stroke scale scores of the individuals show a significant difference according to the Grouped Grit Status (Low, Medium, and High)?

Within the framework of the analysis of the results, it is seen that the stroke levels of the people show a significant difference according to their grit status [*F*(2–196) = 12.94, *p* < 0.01]. In other words, stroke levels vary significantly according to the grit status. When we look at the results of the Scheffe test, which was performed to determine between which groups the differences between the grit groups were, we found that the mean of low stroke (*X* = 2.06) is lower than means of medium stroke (*X  ®*=2.46) and high stroke (*X  ®*=2.69). This finding shows that with the increase in stroke levels of the people, their grit status also increases.

*Research Question 3:* Are there are any effects of stroke components (verbal, non-verbal, valuing, and activities) on grit conditions?

An ordinal logistic regression analysis of link function, logit, was used to determine the effect of stroke components (verbal, non-verbal, valuing, and activities) on the grit state. The results of the parallel curves assumption of the ordinal logistic regression model are given in Table 3. The results in the table reveal that the regression coefficients estimated within the framework of the regression model structured with the logit, the link function, are the same in each category of the dependent variable and the parallel curves assumption is satisfied (*p* > 0.05).

**Table 3:** Test of parallel lines.*

Model	-2 Log likelihood	Chi-square	Df	Sig.
Null hypothesis	382.09			
General	379.57	2.52	4	0.64

The null hypothesis states that the location parameters (slope coefficients) are the same across response categories. *Link function: Logit.

Table 4 also includes the Pearson Chi-square fitting test of the model to demonstrate the suitability of the structured model. When the findings related to the test in question are examined in Table 4, it is seen that the logit-linked ordinal logistic regression model is appropriate (*p* = 0.35 > 0.05). On the other hand, the pseudo *R^2^* values that give information about the relationship between the dependent variable (grit state) and independent variables (verbal, non-verbal, valuing, and activities) were found as Cox-Snell (0.13), Nagelkerke (0.15), and McFadden (0.07). Here, the pseudo *R^2^* values indicate that the stroke components poorly explain the variability in the grit state.

**Table 4:** The results of the fitting test of the model.

Model	Ki-kare	Serbestlik derecesi	P-değeri
Pearson	388.22	378	0.35
Deviance	376.55	378	0.51

Link function: Logit.

Parameter estimates, standard errors and *p* values of the variables in the model are given in Table 5. As a result of the logit-linked ordinal logistic regression analysis resulted in 2 threshold values calculated in the model being statistically significant. These threshold values are used to calculate the probability values of different dependent variable categories.

**Table 5:** Estimated parameter values and significance test results of logit-linked ordinal logistic regression model.

		Estimate	Std. error	Wald	df	Sig.
Threshold	[GRIT = 1,00]	1.731	0.671	6.644	1	0.01
	[GRIT = 2,00]	3.846	0.721	28.498	1	0
Location	Verbal	−0.053	0.259	0.041	1	0.839
	Non_Verbal	0.745	0.282	6.999	1	0.008
	Valuing	−0.01	0.295	0.001	1	0.974
	Activities	0.51	0.265	3.694	1	0.055

As seen in Table 5, non-verbal stroke in the model was the only explanatory variable statistically affecting the grit status. The predicted parameter value of the non-verbal stroke variable, which has a statistically significant effect on the dependent variable, has a positive sign.

When the value of the non-verbal stroke variable, whose estimated parameter value is statistically significant and has a positive sign, is increased by one unit, it will cause an increase in the grit level as much as the parameter value it has. In other words, when the non-verbal stroke score increases by one unit, the grit level will increase by 0.745.

## Discussions

The findings indicate that the relationship between the Grit scale score and the Stroke scale score is moderate, positive, and statistically significant. This implies that when the students’ levels of grit are high, so are their levels of stroke. This finding is consistent with those of Qiao (2022), who claims that a positive psychological quality like grit has a positive relationship with stroke based on earlier research. In other words, students feel motivated and positive about the learning process when the support they get from their teachers matches their grit. Additionally, this supports the findings of Yuan (2022) and Shen and Guo (2022). Therefore, it is not surprising that researchers advocate for the implementation of activities that increase grit.

The second reasearch question investigated whether the Stroke scale scores of the individuals differed significantly according on their Grouped Grit Status (Low, Medium, and High). The results of the investigation suggest that people’s stroke levels differ significantly depending on their grit status. This result indicates that when people’s stroke levels rise, so does their grit. This result was in line with our second hypothesis where it said that the Stroke scale scores of the individuals show a significant difference according to the Grouped Grit Status. This observation is consistent with the findings of Li and Dewaele (2021), who discovered that learners’ grit changes and is not static. For example, if learners enjoy themselves and receive praise and encouragement, their grit is high. When students receive a negative stroke, their grit levels fall between low and medium. Yuan’s (2022) results back up these findings that stroke and grit are associated. It is critical to recognise that reverse psychology should be avoided because it is not always effective. Giving positive feedback to learners encourages them to stay on their path and grow.

The final research question this paper addressed was whether stroke components (verbal, non- verbal, valuing, and activities) had any impact on grit conditions. Yes, there are consequences of stroke components (verbal, nonverbal, valuing, and activities) on grit conditions, according to the hypothesis for this inquiry. The idea was disproved since nonverbal strokes were the only ones that had an impact on grit levels. Smiles or frowns directed at students are nonverbal examples (Pishghadam and Khajavy, 2014). This finding is similar to that of Gholamrezaee and Ghanizadeh (2018), who discovered that nonverbal stroke is more helpful than the other components of stroke in assisting learners in overcoming the challenges they confront during the learning process. Derakhshan et al.’s (2022) study emphasises the superiority of the nonverbal stroke component. These findings indicate that teachers and educators must be well-equipped with all of the necessary information on body language and be psychologically prepared to interact with students in ways other than vocally.

The findings of this work confirm the importance of positive psychology, such as grit, shown in prior research. Duckworth et al. (2007) have long emphasised that grit must be fostered because it is one of the most important predictors of success. When instructors focus on encouraging students, their grit increases and they become more driven than ever to succeed. Grit, in turn, influences the other components of success and longevity. This tells us “none of the interacting variables is entirely stable and that they influence each other” (Li and Dewaele, 2021). As a result, it is critical for future studies to include multiple aspects when studying grit, as it can vary based on the parameters considered. Dewaele and Thirtle (2009), for example, suggest that an individual’s grittiness is influenced by his or her environment, either favourably or adversely.

The present paper has some drawbacks. For starters, as participants in the questionnaire survey self-reported their responses, social desirability might slightly skew their responses. Additionally, while character traits like grit are universal, they may be more highly appreciated and supported in other cultures. As a result, they may take on a slightly different significance when it comes to influencing the emotions and conduct of FL learners. Although the general impacts of grit appear to be universal, we cannot rule out cultural variations.

## Conclusion

As stated at the commencement of this study, positive psychology has gained significant attraction in the twenty-first century. Grit is a component of positive psychology that has garnered additional attention. Considering grit, particularly L2-grit, is such a new notion, it provides researchers with several research opportunities. Students’ stroke is also a novel idea that gained prominence following the development and validation of its scale. However, the relationship between L2-grit levels and students’ stroke was not thoroughly investigated; thus, this work was deemed necessary to address the research gap.

The current study supported earlier findings that grit can alter and can either favourably or adversely correlate with other variables. It was discovered that the L2-grit scale scores and the Stroke scale score have a positive correlation. Additionally, it was discovered that individuals with high, medium or low levels of grit have significantly different stroke rates than those with lower levels. Finally, it was discovered that only nonverbal strokes had a substantial effect on grit levels.

To conclude, this article provides additional proof of the value of grit in the learning process. It also provides valuable information on the linkage between L2-grit and students’ stroke, which should lead to further research in diverse circumstances.

### Pedagogical implications

The findings of the current study have pedagogical implications for EFL instructors and institutions. First of all, it is clear how important grit is and how it affects students’ abilities; as a result, it is crucial that educators and institutions encourage grit. The unwillingness to communicate is one issue that EFL students encounter (Lan et al., 2021). Teachers must inspire their students to face their fears and begin speaking the L2 in order to succeed. Grit “correlates with motivation and other constructs such as self-efficacy, resilience, and hardness,” claim Gyamfi and Lai (2020). Instead of spoon-feeding students everything, EFL teachers and institutions can include instructions on how important effort is in learning a new language.

Additionally, the results of the current study offer language learners helpful advice on how to approach the challenging language learning process (Wang et al., 2021). It encourages students to stop asking common questions like, “How do I learn English,” as by asking this question, students are looking for short cuts. Students of language will value patience and begin approaching the process with an open mind rather than rushing everything once they learn about grit and how it is “passion and perseverance for long-term goals.”

## Data availability statement

The original contributions presented in the study are included in the article/Supplementary material, further inquiries can be directed to the corresponding author.

## Ethics statement

The studies involving human participants were reviewed and approved by the Institute of Graduate Studies and Research Education and Graduate Sciences Center. The patients/participants provided their written informed consent to participate in this study.

## Author contributions

DM, BM, and YK contributed to the conception and design of the study and wrote sections of the manuscript. DM organized the database and wrote the first draft of the manuscript. BM and YK performed the statistical analysis. All authors contributed to the article and approved the submitted version.

## Conflict of interest

The authors declare that the research was conducted in the absence of any commercial or financial relationships that could be construed as a potential conflict of interest.

## Publisher’s note

All claims expressed in this article are solely those of the authors and do not necessarily represent those of their affiliated organizations, or those of the publisher, the editors and the reviewers. Any product that may be evaluated in this article, or claim that may be made by its manufacturer, is not guaranteed or endorsed by the publisher.
